# Defining Elite Zones: A Scoping Review of Body Physique and Body Fat in Elite Athletes

**DOI:** 10.3390/jfmk11010013

**Published:** 2025-12-29

**Authors:** Ximena Martinez-Mireles, Erik Ramírez, José Omar Lagunes-Carrasco, Ricardo López-García, Silvia García, Cristina Bouzas, Rogelio Salas-García, Josep A. Tur

**Affiliations:** 1 Facultad de Salud Pública y Nutrición, Universidad Autónoma de Nuevo León, Monterrey 64460, NL, Mexico; 2 Facultad de Organización Deportiva, Universidad Autónoma de Nuevo León, Monterrey 64455, NL, Mexico; 3 Research Group on Community Nutrition & Oxidative Stress, University of the Balearic Islands-IUNICS, Guillem Colom Bldg, Campus, 07122 Palma de Mallorca, Spain; 4 CIBEROBN (Physiopathology of Obesity & Nutrition), Instituto de Salud Carlos III, 28040 Madrid, Spain; 5 Fundació Institut d’Investigació Sanitària Illes Balears (IdISBa), 07120 Palma de Mallorca, Spain

**Keywords:** elite athletes, body physique, body fat percentage, fat-free mass, fat mass

## Abstract

**Background:** Body physique refers to body size, structure, and composition. PS is used to describe the profile of athletes in different sports. **Aims:** To determine body physique and body fat percentage in elite athletes using the Hattori chart and to identify the elite zone. **Methods:** Scoping review. The search was performed in PubMed, Google Scholar, Ovid Books, CAB eBooks, Clarivate InCites, MyiLibrary, Web of Science, Taylor & Francis Online, Core Collection, and Scopus. The search strategy was “body physique” OR “anthropometric” OR “body composition” AND “elite athlete” OR “athlete” OR “elite”. **Results:** Using indirect methods, elite athletes showed intermediate solid body physique (male) and lean intermediate body physique (female), and 13.6% ± 3.6% (male) and 22.3% ± 2.8 (female) body fat. Using doubly indirect methods, elite athletes showed lean intermediate body physique (male), and intermediate body physique (female), and a percentage of body fat of 13.7% ± 5.2% (male) and of 21.7% ± 4.3% (female) of body fat. **Conclusions:** Hattori’s chart facilitates the visualization of changes in body mass index, fat-free mass index, fat mass index, and percentage of body fat, helping personalize training, monitor composition changes, and guide nutrition programs to optimize performance and health.

## 1. Introduction

Body physique refers to body size, structure, and composition [1] and is used to describe the profile of athletes in different sports [2]. The Heath & Carter [3] somatotype is one of the most common methods for classifying somatotypes, which divides morphology into fat, musculoskeletal development, and relative linearity. Another method is the modified weight somatogram [4], which distinguishes muscular and non-muscular areas. The Phantom proportionality model [5] evaluates body proportions subjectively, without relying on absolute measurements. The Hattori chart [6] used the fat-free mass index (FFMI) and the fat mass index (FMI) to represent body physique, combining the percentage of body fat (BF%), a key component of body physique, and the body mass index (BMI). This last method is flexible and applicable to any population group. All of these methods can be applied to athletes of all sports [7], evaluating both muscle development [8] and body physique [9]. This method is flexible, applicable to any population group, and allows comparisons across sexes, disciplines, and performance levels. Furthermore, unlike the other methods mentioned above, which can mask differences in FMI and FFMI, the Hattori chart separates fat and lean contributions, providing a more precise morphological phenotype into nine categories. Given these advantages, the Hattori method offers clearer interpretability for sport scientists aiming to monitor body composition changes, classify physique types, and identify morphological zones associated with performance.

Morphological optimization is essential to maximize performance and monitor training success [10]. BF% is a crucial performance indicator for elite athletes in sports such as boxing, wrestling, and athletics. In aerobic disciplines such as marathon, low BF% and control of lean mass improves performance and helps to meet weight classes. Conversely, a high BF% may be beneficial in sports where body weight is relative, such as kayaking and canoeing [11]. Methods to measure BF% have evolved, from invasive procedures to more accessible indirect non-invasive techniques. However, despite the advances, differences between methods and the lack of standardization make it difficult to compare their precision [12].

Elite athletes are noted for their superior performance, consistent achievements, and years of experience [13]. Particularly, endurance athletes exhibit better adaptations in anaerobic threshold and VO_2_max [14]. Over time, athletes have shown increases in height and body mass, outpacing trends in the general population. This change has been associated with better performance and economic benefits. However, in some sports, athletes are required to maintain a specific range of body size, which has led to a stable morphology [15].

The evaluation of body physique in athletes is essential to classify and establish differences in terms of size, structure, and composition. This allows precise monitoring of performance, as well as comparisons between positions and other sports, helping to evaluate changes throughout macrocycles and facilitating the monitoring of morphological optimization. These results can be useful as a basis for future research, helping sports professionals to make appropriate decisions to improve the physique and performance of athletes. The evaluation of body physique in elite athletes has been used in fencing, artistic gymnastics, and combat sports to evaluate body composition, weight categories, styles of sport disciplines, and differences between sexes [16,17,18,19,20,21,22,23]. The performance of elite athletes is closely linked to their body physique, especially BF%. Despite biological and adaptive differences in body physique among elite athletes, few studies have summarized BF% data. Research in this area has been scarce lately, focusing on data from athletes prior to the year 2000 [11,16,24,25,26]. However, despite the greater availability of body composition studies in international athletes, the body physique and variation in fat percentage in modern elite athletes of both sexes has been scarcely determined [16]. The novelty of this scoping review lies not in the use of Hattori’s chart for elite athletes, but in its comprehensive synthesis of the available information, identification of gaps in knowledge, and provision of a solid basis for future research on the body physique of elite athletes of both sexes. Therefore, this scoping review aimed to determine body physique and body fat percentage in elite athletes using the Hattori chart [6] and to identify the elite zone.

## 2. Methods

Design. This study was carried out based on the 2018 PRISMA Extension for Scoping Reviews (PRISMA—ScR) guideline [27]. As this study is a scoping review, it was not registered in PROSPERO.

Eligibility criteria. The studies included in this scoping review were published between 1995 and 2024. From this date, indirect methods such as air-displacement plethysmography (ADP) and dual x-ray densitometry (DXA) were more widely used in assessment of body composition [28]. During the analysis of these data, an attempt was made to represent the secular evolution in the morphology of the elite athlete. Articles in English, Portuguese, and Spanish languages were selected, and studies on elite male and female athletes aged 12 to 45 were reviewed. This broad age spectrum was chosen because the highest performance varies widely across sports, occurring mainly in adolescence for several specialized disciplines such as gymnastics, but extending into the late 20s, 30s, or even 40s in endurance and strength events [29,30,31,32,33,34,35]. Articles that did not specify age (*n* = 3) or with open category for age (*n* = 1) were also included. The athletes were elite level, senior, Olympic, first division professionals, world record, world class, national level representatives, black belt, French Rating Scale of minimum 7a, non-professional sports with at least 10 years of experience, TOP ranking 10th at the national or international level, or high-level collegiate (world class, national representatives, or first division of the National Collegiate Athletics Association or first league of universities) [36].

The indirect methods used to assess body composition were hydrostatic weighing, air-displacement plethysmography (ADP) and dual energy X-ray absorptiometry (DXA). The doubly indirect methods considered were bioelectrical impedance analysis (BIA) with a minimum of four electrodes, ultrasounds, and anthropometry with equations derived from two-, three-, and four-compartment models. In papers that showed measurements with several methods, priority was given to data derived from the following techniques in this order: ADP, DXA, hydrostatic weighing, BIA, ultrasound, and anthropometry. Studies that calculated BF% with at least three skinfolds, including those for calculating body density, were also considered. When a study did not report the BF%, this value was determined as: BF% = fat mass (kg) * 100/body mass (kg). Finally, body composition data in basal, initial, or pre-competition periods were considered.

Articles that featured male and female athletes at the amateur, recreational, Paralympic, preschool, and school level (under 12 years of age), second and third division athletes, those with less than 9.9 years of experience, extreme sports, and winter sports were excluded. Winter sports were excluded because their environmental and physiological demands such as cold exposure and thermoregulation, as well as equipment constraints create morphological patterns not comparable to those of non-winter sports. Including winter sports would have reduced conceptual and methodological consistency in the analysis.

However, an article on an amateur athlete with a national TOP ranking of 7 was included. Articles from elite athletes that did not specify sex, sport practiced, reported median, or reported grouped body composition data were not considered. Also, those who used formulas to obtain BF% with less than two skinfolds and who did not present fat mass data (kg) were not considered.

Information sources. The literature search was carried out in the electronic databases Medline via PubMed, Google Scholar, Books Ovid, CAB eBooks, Clarivate InCites, MyiLibrary, Web of Science, Taylor & Francis Online, Core Collection, and Scopus. All studies from 1995 to 2024 were filtered. The first author conducted the search strategy and then discussed them with all authors. To conduct a more comprehensive scoping review, within the selected articles, other potential references were identified through manual searching, allowing them to add relevant articles not considered in the initial search.

Search. The search strategy for each electronic database included the terms “physical status” OR “anthropometric” OR “body composition” AND “elite athlete” OR “athlete” OR “elite”. Additionally, article references from other reviews were manually retrieved.

Selection and sources of evidence. The first author reviewed the articles in duplicate, and the second author performed a review to ensure that they met all inclusion criteria. The authors then examined 233 data from elite athletes and sought information on each body composition team that met the eligibility criteria. In articles that used published equations to predict BF%, the original sources were consulted to verify the correct transcription of the equations used. Three meetings were held between May and August (2024) to review the database. The review resulted in deleting 111 articles. The data from the studies collected included the following: last name of the first author, year published, title of the article, sport, type of sport, athlete level, specialty or position, body composition method, skin caliper, equation used, software version or model of the equipment (ADP, DXA, and BIA,), age, sex, height (cm), body mass (kg), BF%, and/or fat mass (FM).

Data collection process. Following the deleted articles, a meeting was held with all authors to discuss data elements relevant to a scoping review. The data for the development of the Hattori chart [6] were body mass (kg), height (m), BMI (kg/m^2^), and BF%. To prepare the body physique tables, the Nutri Solver^®^ software v.1 was used [36]. As quality control, the fat mass index (FMI) and fat-free mass index (FFMI) values were compared between the software and those calculated using Excel^®^. For the analysis of BF% and BMI, the D’Agostino Kurtosis descriptive statistical test was carried out to evaluate whether the data were normally distributed.

Data elements. To develop the results tables of this study, information on the country of origin or type of competition, athlete level, first author, year of publication, age, body mass, height, BMI, FMI, and FFMI were extracted from the publications, and the classification of body physique according to Hattori’s chart [7] was followed. To prepare the body physique graphs, FMI, FFMI, and athlete type data were used; moreover, the weighted average of BMI and BF% were obtained.

The elite zone was determined by integrating four variables: BMI, BF%, FMI, and FFMI. The FMI and FFMI were used to position athletes within the Hattori chart, as these indices independently represent fat and lean tissue adjusted for height and therefore define the compositional basis of the physique categories. BMI and BF% were analyzed alongside FMI and FFMI because they are consistently reported across studies using indirect and doubly indirect methods, enabling the synthesis of heterogeneous datasets. Moreover, BMI and BF% define the external phenotype in Hattori’s conceptual framework and allow the construction of interpretable boundaries that reflect the distribution of elite athletes. The mean and standard deviation of BMI and BF% were calculated across all included samples, and the ±1 SD and ±2 SD limits were used to outline the central morphology of elite athletes within the FMI–FFMI coordinate space. This combined approach provides both compositional precision and practical interpretability for sport scientists.

Synthesis of results. Studies were grouped by doubly indirect methods and indirect methods (male and female elite athletes), which were presented in tables separately by method and sex. To graph the results, the NCSS-8 program version 8.0.24, (Number Cruncher Statistical Systems, Kaysville, UT, USA) was used [37]. Body physique categories were derived from the mean and standard deviation (SD) of BMI and FFMI of adults aged 20.0 to 29.9 years [38] based on the third survey of the National Health and Nutrition Examination Survey (NHANES III). This age was selected since the elite athletes in the current study showed a mean age of 24.0 ± 5.4 years. The equation to determine BMI was body mass (kg)/height (m)^2^. In accordance with Hattori’s methodology [39], fat mass and fat-free mass were adjusted by the subject’s height to obtain body composition indices: FMI = fat mass (kg)/height (m)^2^ and FFMI = fat-free mass (kg)/height (m)^2^. The FMI and FFMI cut-off points were calculated from the mean ±1 SD. The FMI was placed on the Y-axis and the FFMI on the *X*-axis. The resulting categories are described in Table 1.

The combination of the coordinates of the *X*-axis (FFMI) and *Y*-axis (FMI) resulted in nine categories that determined different body physique morphotypes according to Hattori et al. [39] (Table 2).

The weighted means of BF% and BMI were used only to plot the group’s central point in the Hattori chart. To draw the constant BF% lines, BF% was derived from the mathematical relationship between FMI and FFMI using Hattori’s equation BF% = FMI/(FMI + FFMI) (100) [39]. This equation was applied exclusively to generate the lines in the chart and not to estimate the BF% of individual athletes, whose values were extracted directly from the original studies. In the second chart, the elite zone for athletes was represented as ±1 SD for BMI and BF% (68.2% of athletes). The second elite zone, ±2 SD of BF% and BMI, encompassed 95.45% of the population. To prepare the descriptive tables of the body physique categories, the weighted means of BMI, BF%, FMI, and FFMI were also determined. The risk of bias in the studies, summary measures, and additional analyses of the included studies was not assessed [27].

## 3. Results

**Figure 1:**
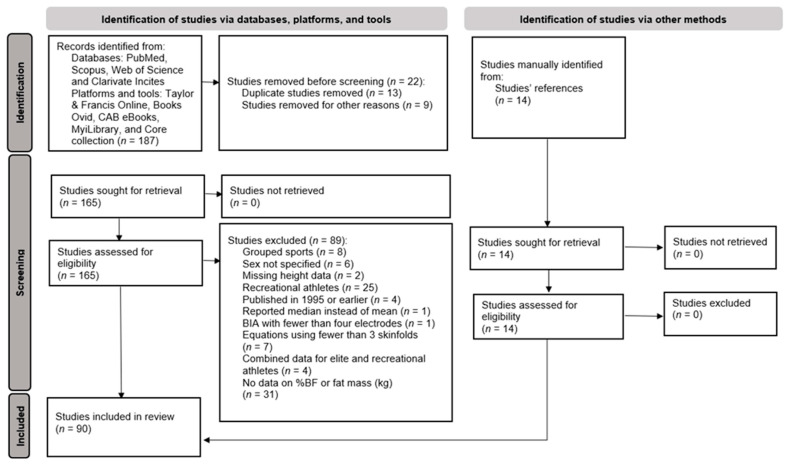
PRISMA flow-chart of the process.

From the initial searched articles (*n* = 187), 14 articles were added through manual searching in review studies, resulting in 201 obtained articles. Duplicate studies (*n* = 13) and keywords (*n* = 9) were screened and deleted. Next, 165 articles were evaluated for eligibility; 89 studies were excluded and 90 were included for the final review. Figure 1 shows the PRISMA flow-chart for the inclusion, exclusion, and removal of studies.

The main characteristics of the selected studies were presented with the following data: sport practiced, elite level, reference, age (years), height (meters), BMI (kg/m^2^), BF%, FMI (kg/m^2^), FFMI (kg/m^2^), and body physique. Likewise, the body composition results of elite athletes were shown with a total of 232 data points. Data from indirect methods in male athletes (*n* = 43) are shown in Table 3. The least used was hydrostatic weighing (*n* = 1), followed by ADP (*n* = 10) and DXA (*n* = 32). Data on elite female athletes (*n* = 18) are shown in Table 4. All data were obtained by DXA. See Supplemental Figures S1 and S2 for males and females, respectively.

Table 5 shows the body composition results of the elite male athletes (*n* = 115) that were measured with doubly indirect methods such as ultrasound (*n* = 3), BIA (*n* = 22), and anthropometry (*n* = 90). Table 6 shows articles from elite female athletes (*n* = 56). The methods reported were ultrasound (*n* = 3), BIA (*n* = 17), and anthropometry (*n* = 36). In total, combining indirect and doubly indirect methods, data from 232 athletes from 61 sports were considered. The risk of bias of the studies and additional analyses of the included studies were not assessed [27].

Body composition assessment using doubly indirect methods was applied in 65 papers, of which 47 used anthropometry. The equations most used in articles to calculate body composition in elite male athletes were Jackson and Pollock [63] (*n* = 9), followed by Durnin and Womersley [66] (*n* = 8) and Withers et al.’s unpublished data [10] (*n* = 7). In elite female athletes, the most used equations were Jackson et al. [124] (*n* = 7), Durnin and Womersley [66] (*n* = 5), Yuhasz [87] (*n* = 3), and Withers et al. [135] (*n* = 3).

The body physique of male elite athletes was lean intermediate with a BF% of 14.1 ± 5.4% (elite zone ±1 SD for BF%: 8.7% to 19.5%; BMI: 21.3 to 28.7 kg/m^2^). Similarly, female elite athletes exhibited lean intermediate physique with a BF% of 21.8 ± 4.1% (elite zone ±1 SD for BF%: 17.7% to 25.9%; BMI: 20.5 to 23.6 kg/m^2^).

A predominantly intermediate solid body physique (41.9%), followed by lean intermediate (23.3%) and lean solid (20.9%), adipose solid (9.3%), and intermediate (4.7%) physiques, was defined using indirect methods in 26.5 ± 5.2-year-old elite male athletes. The average BF% was 13.6% ± 3.6. Dominant body physiques of lean intermediate (38.9%), intermediate (33.3%), intermediate solid (22.2%), and lean solid (5.6%) were defined in 23.4 ± 5.6-year-old elite female athletes. The BF% in elite female athletes, measured by indirect methods, was 22.3% ± 2.8.

Using doubly indirect methods, the mean age of the elite male athletes was 23.8 ± 5.7 years. They showed a predominant body physique of lean intermediate (42.6%), followed by intermediate (27.0%), lean solid (14.8%), intermediate solid (12.2%), adipose solid (2.6%), and lean slender (0.9%). The average BF% was 13.7% ± 5.2. The elite female athletes assessed with the doubly indirect method had an average age of 23.3% ± 4.8 and an intermediate dominant body physique (44.6%), followed by lean intermediate (41.1%), lean solid (7.1%), and intermediate solid (7.1%). The mean BF% was 21.7% ± 4.3.

The elite zone of the athletes was defined as ±1 SD and ±2 SD for BF% and BMI. With indirect methods, the elite zone of BF% in male athletes at ±2 SD was from 6.4% to 20.8% and for BMI, from 19.8 to 33.8 kg/m^2^. In female athletes at +2 SD, the elite zone of BF% was 16.7% to 27.9% and for the BMI of 18.4 it was 25.2 kg/m^2^. With the doubly indirect methods, the elite zone of BF% in male athletes (±2 SD) was from 3.3% to 24.1% and for BMI, from 19.5 to 28.3 kg/m^2^.

In female athletes, the elite zone at ±2 SD of BF% was from 13.1% to 30.3% and for BMI, from 19.2 kg/m^2^ to 25.2 kg/m^2^. The elite zone at +2 SD encompassed five morphotypes in male and female athletes of indirect and doubly indirect methods: intermediate slender, intermediate, intermediate solid, lean solid, and lean intermediate. None of the elite athletes were in the lean intermediate quadrant, even though the elite zone encompassed this morphotype.

A description of the different categories of body physique for indirect and doubly indirect methods in elite athletes was made. Applying indirect methods, the predominant body physique in male athletes was intermediate solid (BMI 30.1 ± 3.4; BF% 15.9 ± 2.6; BMI 4.8 ± 1.2; FFMI 25.2 ± 2.5) (Table 7). In female elite athletes assessed with indirect methods, the dominant body physique was lean intermediate (BMI 20.2 ± 0.9; BF% 20.9 ± 1.7; 4.1 ± 0.6; FFMI 16.0 ± 0.7) (Table 7). With the doubly indirect methods, in elite male athletes, the predominant body physique was lean intermediate (BMI 22.2 ± 1.1; BF% 9.8 ± 2.1; BMI 2.1 ± 0.5; FFMI 20.0 ± 1.0) (Table 8). In female elite athletes assessed with doubly indirect methods, the dominant body physique was intermediate (BMI 22.9 ± 0.8; BF% 24.4 ± 2.4; IMG 5.5 ± 0.7; FFMI 17.2 ± 0.5) (Table 8). See Supplemental Figures S3 and S4 for males and females, respectively.

## 4. Discussion

This study shows that male elite athletes measured using indirect methods predominantly had an intermediate solid body physique. In female elite athletes, the dominant body physique was lean intermediate. Using the doubly indirect methods, male elite athletes had a lean intermediate body physique, whereas female elite athletes had an intermediate body physique.

The variability in indirect and doubly indirect methods to estimate BF% varies between ethnic groups due to differences in subcutaneous fat distribution [140]. Accordingly, this review reported the methods separately. Moreover, the heterogeneity and accuracy of body composition methods may affect the assessment of body physique, mainly due to the limitations of each method, and then affect the classification of athletes in categories according to those described in Table 2.

In a study conducted in young adults, several methods were compared, including hydrostatic weighing, BIE (doubly indirect method), and DXA (indirect method); the last is considered the gold standard. It was observed that hydrostatic weighing (indirect method) showed the highest data in women, overestimating BF% by 2.9%, followed by BIE (doubly indirect method), which overestimates it by 2.7%. Anthropometric equations (doubly indirect method) can show significant variations in BF% ranging between 8.0% and 29.0% in women, and between 6.0% and 29.0% BF in men. Therefore, the choice of any equation must be made with caution, considering its precision and the characteristics of the population to be measured [141].

In the same discipline, in this case ballet, differences in BF% of 6.2% were observed between hydrostatic weighing and DXA (indirect methods) and BIE (doubly indirect method) [59]. Differences have also been found in elite gymnasts when comparing methods such as DXA with anthropometry (doubly indirect method) with a difference of 3.0% [57]. But even between DXA equipment with differences between pencil beam and fan beam, differences of 0.9% were found in Australian football players [46]. These discrepancies underline the importance of considering the methodological error that affects the comparison of results. As has been generally observed, DXA (indirect method) is used as a precise method for evaluating body composition in athletes [12,142,143,144].

Male elite athletes evaluated with indirect methods are mostly classified as intermediate solid, characterized by a high FFMI (25.2 ± 3.0 kg/m^2^), a moderate FMI (4.8 ± 1.2 kg/m^2^), and a mean BF% of 15.9 ± 2.6, classified as “Fair” according to the American College of Sports Medicine [145]. This body physique includes American football players, judokas, water polo players, and rugby players, who require strength and power, although they showed differences depending on the specific demands of each sport. In American football, positions could change according to strength and body mass, while speed and agility are more important in other positions such as linebackers and running backs [146]. In sports such as rugby and water polo, higher aerobic ability is required due to continuous physique activity, while judokas tended to have a low BF% and high muscle mass, which favors their performance [147]. Strength athletes are characterized by high body mass and bone mineral content, with a high BMI and a significant amount of fat mass [42,56,148,149,150,151]. The adipose solid category includes offensive and defensive linemen, who have a large size and high level of strength to absorb impacts [50,152,153] Sumo wrestlers are characterized by their high body mass, fat, and muscle [16,154,155]. Endurance sports, and those divided into weight categories tend to have the lowest BF% values, while in sports where body size is advantageous, there may be higher BF%.

In female elite athletes measured with indirect methods, the main body physique was lean intermediate, its main characteristics were a moderate FFMI (16.0 ± 0.7 kg/m^2^) and a low FMI (4.1 ± 0.6) with a mean BF% of 20.9% ± 1.7, within the “Fair” classification [145]. In this category are ballet dancers, soccer players, field runners, and gymnasts, who, despite their morphological differences, have a lower BMI that highlights their muscle tone. Ballet dancers have a higher BF% and lower muscle mass than gymnasts, but they stand out for their biomechanical and balanced skills. Dancers, field runners [156] and gymnasts [157] face health problems associated with low energy availability and bone mineral density. Female soccer players showed better biomechanical patterns and bone health in general [158,159,160].

Applying the doubly indirect methods, male elite athletes showed a predominantly lean intermediate physique, with a moderate FFMI (20.0 ± 1.0 kg/m^2^), a low FMI (2.1 ± 0.5 kg/m^2^), and a BF% of 9.8 ± 2.2, classified as “Excellent” [145]. This body physique, which emphasizes muscle definition and lean appearance, includes volleyball players, basketball players, boxers, taekwondo athletes, rowers, marathon runners, triathletes, sprinters, gymnasts, sprint swimmers, and other athletes who benefit from a low BF%. These characteristics improve agility, speed, endurance, and ability to change direction rapidly [121,161,162,163,164,165,166]. In the adipose solid body physique, athletes showed a mean FFMI of 28.1 ± 2.2 kg/m^2^, an FMI of 10.8 ± 0.3 kg/m^2^, and a BF% of 27.9 ± 2.1. This group includes heavy-weight powerlifters, where high fat-free mass is crucial to maximize squat, bench press, and deadlift performance [167,168,169]. However, high FMI can negatively affect relative strength [169].

In female elite athletes measured using doubly indirect methods, an intermediate body physique predominated, characterized by a moderate FFMI (17.2 ± 0.5 kg/m^2^) and FMI (5.5 ± 0.7 kg/m^2^) and a BF% of 24.4% ± 2.4 of poor classification [145]. Within this morphotype, open water swimmers benefit from higher BF%, which provides them with buoyancy and reduces water resistance; they tend to be smaller and lighter than competitive pool swimmers, which helps them in their endurance performance [170,171]. Female basketball and tennis players share several physical characteristics, including a lean body composition, high agility, significant aerobic and anaerobic capacity, and strong coordination and movement skills. These attributes are essential for the fast and dynamic movements required in both sports [172,173]. Female kayakers generally have larger body sizes, including higher body mass and height. This larger body size is associated with the specific demands of their sport [174]. There are also softball players, who tend to be taller and have larger biacromial and iliocristal diameters of the femur, as well as a greater femoral bicondylar diameter [175].

Comparing Hattori’s chart [39] with Heath and Carter’s [3] somatotype, the mesomorphic component, which reflects musculoskeletal robustness [176], is related in Hattori’s chart to the lean solid body physique. Ectomorphy corresponds to lean slender, and endomorphy to adipose intermediate. Studies in elite athletes [177] showed similarities between mesomorphy and the categories of Hattori’s chart. While somatotype focuses on a single component, Hattori’s chart classifies mesomorphic into four types, providing a more precise evaluation. However, few studies categorized body physique in elite or recreational athletes using the categories proposed by Hattori [6,39,178]. Among the studies published using this methodology, most of them focused on high or low FMI or FFMI [16,17,18,19,20,21,22,23]. In summary, the Hattori chart is useful for classifying body physique and monitoring changes in body composition, offering comparative references. However, it is suggested that further studies be conducted across disciplines and among elite female athletes.

In this study, the elite zone was identified in male athletes using indirect methods with BF% between 6.4% and 20.8% and BMI from 19.8 kg/m^2^ to 33.8 kg/m^2^. In female elite athletes, the range was 16.7% to 27.9% for BF% and 18.4 kg/m^2^ to 25.2 kg/m^2^ for BMI. For the doubly indirect methods, the male elite zone was established with a BF% of 3.3% to 24.1% and a BMI of 19.5 kg/m^2^ to 28.3 kg/m^2^, while in women it was from 13.1% to 30.3% for BF% and from 19.2 kg/m^2^ to 25.2 kg/m^2^ for BMI. The morphotypes analyzed at ±2 SD included categories such as adipose solid, intermediate slender, intermediate, and intermediate solid, and lean slender, lean intermediate, and lean solid.

It was observed that SDs reflect variations in the athletes’ body physique, depending on the sport, and even the position or category within the same discipline. For example, gymnasts fall into the lean category, while sumo wrestlers and football linemen fall into the adipose solid category. These variations optimize sports performance according to the specific demands of the sport, such as judo, boxing, or rowing, where body composition directly influences performance [177].

To our knowledge, only one study differentiated the elite zone into categories for sumo wrestlers compared to the general population [16]. Identifying the elite zone could facilitate the monitoring of athletes in the different disciplines. The elite zone is a universal phenomenon experienced by almost all elite athletes, and it has been described as the pinnacle of achievement for an athlete and characterizes a state in which an athlete performs to the best of his or her ability [175,176]. The ±1 SD (±2 SD) zone would help identify differences between sports and optimize body composition to improve training cycles and sports performance or take advantage of biomechanical advantages, obtaining the highest performance in each sport discipline.

Identifying the elite zone (BMI ± 2 SD and BF% ± 2 SD) offers sport scientists a reference for monitoring athletes’ morphological changes throughout a training cycle. Athletes whose values fall outside this zone can be evaluated to determine whether the deviation reflects desired adaptations (e.g., increases in muscle mass) or potential issues such as excessive fat gain, low energy availability, or loss of lean mass. The elite zone can guide nutritional planning, training adjustments, and return-to-play decisions. In weight-category or aesthetic sports, the ±1 SD range may help identify when an athlete is approaching thresholds associated with increased injury risk or reduced energy availability, while the ±2 SD boundaries can serve as upper and lower limits beyond which performance may be compromised.

### Strengths and Limitations of the Study

The main novelty of this study lies in the breadth of the review or in the comprehensive identification of the “elite zone” across a wider spectrum of sports and genders. Frisancho’s [38] cut-off points for ages 20 to 29.9 years were chosen due to the average age of the athletes evaluated. Although this reference is used as a growth curve because it is derived from the US NHANES surveys, it cannot adequately represent the body size and composition of athletes or all ethnicities. Elite athletes generally have lower body fat percentages compared to the general population, with significant variations by sport. However, this reference can be used as a basis to allow comparison of studies in which variables such as somatotype are used.

Furthermore, each method of estimating body composition has limitations that can influence the classification of athletes. Hydrostatic weighing (indirect method) appears to be unsuitable for athletes who focus on strength due to its demanding technique, and DXA (indirect method) has variations in manufacturers’ algorithms and differences between pencil and fan beams [174]. BIA (doubly indirect method) can be affected by factors such as limb length and hydration [179]. Ultrasound (doubly indirect method), although useful, requires considerable skill and is not standardized [174]. ADP and anthropometry (doubly indirect method) also have limitations in their accuracy. Anthropometry (doubly indirect method) is less precise than DXA (indirect method) and requires specific equations to avoid significant errors in athletes [174,180]. Although the aforementioned methods such as hydrostatic weighing, ADP, and DXA (both indirect methods) are considered reference methods for evaluating body composition in athletes, they are not widely accessible in most evaluation centers. Another limitation is that indirect methods (e.g., DXA) have higher precision than those that are doubly indirect (e.g., BIA).

Regarding anthropometric equations, the algorithm most used to predict BF% in elite athletes was previously proposed [63,124]. Although it does not specifically include athletes, its popularity is broad. Many of the equations reported by the studies are not generalizable to all athletes, since they are not a main part of the sample or are only specific to certain disciplines. Each equation is specific to the population studied, and the structure, body composition, and exercise habits of the population from which they are derived.

## 5. Conclusions

Body physique measured with indirect methods was intermediate solid in male elite athletes, and lean intermediate in elite female athletes. Body physique measured using doubly indirect methods was lean intermediate in male elite athletes, and intermediate in female elite athletes. Hattori’s chart facilitates the visualization of changes in body mass index, fat-free mass index, fat mass index and percentage of body fat, helping personalize training, monitor composition changes, and guide nutrition programs to optimize performance and health. Future research could apply Hattori’s method to differentiate athletes’ body composition by category, specialty, position, and training period.

## Figures and Tables

**Table 1:** Categorization of the fat mass index (FMI) and free-fat mass index (FFMI) according to the body physique classification.

Fat Mass Index (FMI) in kg/m^2^			
Classification	Lean	Intermediate	Adipose
Women	≤4.6	4.7–13.1	≥13.2
Men	≤3.2	3.3–8.7	≥8.8
**Fat-Free Mass Index (FFMI) in kg/m^2^**			
**Classification**	**Slender**	**Intermediate**	**Solid**
Women	≤13.9	14.0–18.2	≥18.3
Men	≤16.4	16.5–21.5	≥21.6

Categorization of the fat mass index (FMI) on the Y-axis and the fat-free mass index (FFMI) on the X-axis of the Hattori chart [6,39]. Cutoff points from 20.0 to 29.9 years based on ±1 SD from Frisancho [38].

**Table 2:** Nomenclature of the nine categories of body physique.

Level	Morphotype
1	Adipose slender
2	Adipose intermediate
3	Adipose solid
4	Intermediate slender
5	Intermediate
6	Intermediate solid
7	Lean slender
8	Lean intermediate
9	Lean solid

Intermediate: level suggested when there is a balance between FMI and FFMI. Adapted from Hattori et al. [39].

**Table 3:** Body composition in elite male athletes with indirect methods.

Sport	Reference	*n*	Method	Age, Years	Height, m	BMI, kg/m^2^	BF%	FMI, kg/m^2^	FFMI, kg/m^2^	Body Physique
Sumo, Japan national	[16]	36	Hydrostatic weighing	21.10	1.791	36.5	26.2	9.6	26.9	Adipose solid
Soccer, Argentina first national division	[40]	42	DXA (Lunar DPX-L)	23.20	1.765	24.5	12.2	3.0	21.5	Intermediate solid
American football, NFL Position: running back	[41]	4	ADP	No	1.8	29.8	7.3	2.2	27.6	Lean solid
American football, NFL Position: offensive lineman	[41]	11	ADP	No	1.93	37.6	25.1	9.4	28.2	Adipose solid
American football, NFL Position: quarter-back	[41]	2	ADP	No	1.92	28.3	14.6	4.1	24.1	Intermediate solid
American football, NFL Position: wide receiver	[41]	7	ADP	No	1.8	26.4	8.1	2.1	24.3	Lean solid
American football, NFL Position: tight end	[41]	4	ADP	No	1.94	30.7	15.1	4.6	26.1	Intermediate solid
American football, NFL Position: line-backer	[41]	5	ADP	No	1.86	31.2	15.7	4.9	26.3	Intermediate solid
American football, NFL Position: defensive lineman	[41]	7	ADP	No	1.9	35.1	18.5	6.5	28.6	Intermediate solid
American football, NFL Position: defensive back	[41]	11	ADP	No	1.79	27.2	6.3	1.7	25.5	Lean solid
American football, NFL Position: kicker	[41]	2	ADP	No	1.91	26.1	11.4	3.0	23.1	Lean solid
Water polo, TOP 4 World Championship 2003	[42]	19	DXA (Lunar DPX)	25.50	1.84	26.8	16.8	4.5	22.3	Intermediate solid
Judo, national from Portugal	[43]	27	DXA (QDR-4500)	23.20	1.76	23.5	12.1	2.8	20.7	Lean solid
Judo, black belt	[44]	27	DXA (QDR 4500A)	22.20	1.76	23.2	11.7	2.7	20.5	Lean solid
Fencing, national from Kuwait	[45]	15	ADP	21.50	1.75	23.2	13.9	3.2	20.0	Lean intermediate
Flat jockeys, England national	[46]	19	DXA (Hologic)	27.00	1.67	20.1	13.0	2.6	17.5	Lean intermediate
Jump jockeys, national from England	[46]	18	DXA (Hologic)	25.00	1.75	21.3	11.5	2.5	18.9	Lean intermediate
Latin dance, TOP 6 in the world	[47]	7	DXA (Lunar DPX-IQ)	21.50	1.75	22.9	3.4	0.8	22.1	Lean solid
Standard dance, TOP 6 in the world	[47]	12	DXA (Lunar DPX-IQ)	26.70	1.83	21.6	11.2	2.4	19.2	Lean intermediate
Ten dance, TOP 6 in the world	[47]	11	DXA (Lunar DPX-IQ)	19.40	1.8	22.3	14.9	3.3	19.0	Intermediate
Australian football, Australia	[48]	47	DXA (Prodigy)	22.70	1.86	24.4	8.5	2.1	22.3	Lean solid
Australian football, Australia	[48]	47	DXA (Lunar DPX-IQ)	22.70	1.86	24.4	7.6	1.9	22.5	Lean solid
Rugby players, Australia national Position: forward	[49]	20	DXA (Hologic Discovery A)	25.40	1.91	30.6	14.2	4.3	26.3	Intermediate
Rugby players, Australia national Position: backs	[49]	17	DXA (Hologic Discovery A)	25.40	1.82	27.7	10.7	3.0	24.7	Lean solid
American football, NFL Position: safety	[50]	69	DXA (Lunar iDXA)	33.00	1.822	27.4	12.1	3.3	24.0	Intermediate solid
American football, NFL Position: receiver	[50]	56	DXA (Lunar iDXA)	33.00	1.857	27.3	12.5	3.4	23.9	Intermediate solid
American football, NFL Position: running back	[50]	36	DXA (Lunar iDXA)	33.00	1.815	32.0	16.0	5.1	26.9	Intermediate solid
American football, NFL Position: tight end	[50]	31	DXA (Lunar iDXA)	33.00	1.929	30.6	16.8	5.1	25.5	Intermediate solid
American football, NFL Position: line-backer	[50]	55	DXA (Lunar iDXA)	33.00	1.867	31.5	17.0	5.4	26.2	Intermediate solid
American football, NFL Position: kicker	[50]	19	DXA (Lunar iDXA)	33.00	1.874	28.0	19.2	5.4	22.6	Intermediate solid
American football, NFL Position: quarter-back	[50]	22	DXA (Lunar iDXA)	33.00	1.885	29.2	19.6	5.7	23.4	Intermediate solid
American football, NFL Position: offensive lineman	[50]	65	DXA (Lunar iDXA)	33.00	1.928	37.9	28.8	10.9	27.0	Adipose solid
American football, NFL Position: defensive lineman	[50]	58	DXA (Lunar iDXA)	33.00	1.909	36.5	25.2	9.2	27.3	Adipose solid
England national cricket fast bowlers	[51]	12	DXA (Lunar iDXA)	22.60	1.87	24.3	17.4	4.2	20.1	Intermediate
American football, first division NCAA	[52]	57	DXA (Hologic)	19.50	1.86	31.1	19.3	6.0	25.1	Intermediate solid
Judo, Olympic level	[53]	17	DXA (Lunar Prodigy)	No	1.76	26.1	14.5	3.8	22.3	Intermediate solid
Box, Olympic level	[53]	15	DXA (Lunar Prodigy)	No	1.71	23.1	9.1	2.1	21.0	Lean intermediate
Wrestling, Olympic level	[53]	14	DXA (Lunar Prodigy)	No	1.77	24.3	13.1	3.2	21.1	Lean intermediate
Taekwondo, Olympic level	[53]	10	DXA (Lunar Prodigy)	No	1.8	21.0	8.8	1.8	19.2	Lean intermediate
Soccer, first division from Spain	[54]	40	DXA (Hologic QDR)	16.67	1.74	22.0	11.86	2.6	19.4	Lean intermediate
Rugby, New Zealand national Position: forward	[55]	23	DXA (Hologic Discovery A)	27.2	1.9	32.3	17.8	5.7	26.5	Intermediate solid
Rugby, national from New Zealand Position: backs	[55]	16	DXA (Hologic Discovery A)	25.7	1.83	28.6	14.8	4.2	24.4	Intermediate solid
Strongman, international	[56]	18	DXA (Lunar iDXA)	33.00	1.87	43.7	18.7	8.2	35.5	Intermediate solid

Abbreviations: ADP: air-displacement plethysmography; BMI: body mass index; DXA: dual x-ray densitometry; FMI: fat mass index; FFMI: fat-free mass index; NCAA: National Collegiate Athletic Association; and NFL: National Football League; FMI and FFMI are derived from height-adjusted values. BF% values were extracted from original studies when available. When BF% was not reported, values were estimated from the fat mass or lean mass provided in the article using proportional calculations based on the reported components.

**Table 4:** Body composition in elite female athletes with indirect methods.

Sport	Reference	*n*	Method	Age, Years	Height, m	BMI, kg/m^2^	BF%	FMI, kg/m^2^	FFMI, kg/m^2^	Body Physique
Artistic gymnastics, national from United States	[57]	11	DXA (Lunar DPX-L)	16.50	1.64	19.0	12.36	2.3	16.6	Lean intermediate
Bodybuilding, Australia national 1998 and 1999	[58]	5	DXA (Lunar DPX-L)	35.60	1.674	21.6	9.7	2.1	19.5	Intermediate solid
Ballet, professional	[59]	1	DXA	35.00	1.682	17.5	15.9	2.8	14.7	Lean intermediate
Handball, national from Italy Position: goalkeeper	[60]	7	DXA (Hologic QDR Explorer W)	24.00	1.69	26.2	29.7	7.8	18.4	Intermediate solid
Handball, national from Italy Position: defender	[60]	14	DXA (Hologic QDR Explorer W)	23.20	1.71	23.2	25.1	5.8	17.3	Intermediate
Handball, national from Italy Position: pivot	[60]	4	DXA (Hologic QDR Explorer W)	23.70	1.67	23.9	22.7	5.4	18.5	Intermediate solid
Handball, national from Italy Position: wing	[60]	18	DXA (Hologic QDR Explorer W)	21.80	1.65	22.4	24.4	5.5	16.9	Intermediate
Standard dance, TOP 6 in the world	[47]	12	DXA (Lunar DPX-IQ)	25.30	1.71	19.6	20.9	4.1	15.5	Lean intermediate
Ten dance, TOP 6 in the world	[47]	11	DXA (Lunar DPX-IQ)	19.00	1.67	19.9	22.6	4.5	15.4	Lean intermediate
Latin dance, TOP 6 in the world	[47]	7	DXA (Lunar DPX-IQ)	21.10	1.63	20.1	22.6	4.5	15.6	Lean intermediate
Soccer, national team from Denmark	[61]	27	DXA (Prodigy Oracle)	24.40	1.72	21.7	20.21	4.4	17.3	Lean intermediate
Judo, Olympic level	[53]	7	DXA (Lunar Prodigy)	No	1.66	24.7	24.9	6.2	18.6	Intermediate solid
Box, Olympic level	[53]	17	DXA (Lunar Prodigy)	No	1.64	22.5	22	5.0	17.6	Intermediate
Wrestling, Olympic level	[53]	5	DXA (Lunar Prodigy)	No	1.63	24.7	21.6	5.3	19.4	Intermediate solid
Taekwondo, Olympic level	[53]	9	DXA (Lunar Prodigy)	No	1.68	21.2	23.1	4.9	16.3	Intermediate
Cross country, first NCAA division	[62]	42	DXA (GE Lunar iDXA)	19.50	1.66	19.9	22	4.4	15.5	Lean intermediate
Lacrosse, first NCAA division	[62]	32	DXA (GE Lunar iDXA)	19.70	1.66	23.1	23.7	5.5	17.6	Intermediate
Swimming, first NCAA division	[62]	28	DXA (GE Lunar iDXA)	19.30	1.71	22.4	23	5.2	17.3	Intermediate

Abbreviations: DXA: dual x-ray densitometry; FMI: fat mass index; FFMI: fat-free mass index; and NCAA: National Collegiate Athletic Association. FMI and FFMI are derived from height-adjusted values. BF% values were extracted from original studies when available. When BF% was not reported, values were estimated from the fat mass or lean mass provided in the article using proportional calculations based on the reported components.

**Table 5:** Body composition of elite male athletes with doubly indirect methods.

Sport	Reference	*n*	Method	Age, Years	Height, m	BMI, kg/m^2^	BF%	FMI, kg/m^2^	FFMI, kg/m^2^	Body Physique
Archery, national from Puerto Rico	[24]	7	A [63]	19.60	1.69	23.3	18.5	4.3	19.0	Intermediate
Bowling, national from Puerto Rico	[24]	22	A [63]	32.20	1.73	26.9	23.4	6.3	20.6	Intermediate
Road cycling, national from Puerto Rico	[24]	40	A [63]	20.20	1.73	22.1	10.6	2.3	19.8	Lean intermediate
Fencing, national from Puerto Rico	[24]	8	A [63]	19.40	1.74	23.0	14.1	3.2	19.7	Lean intermediate
Sports shooting, national from Puerto Rico	[24]	7	A [63]	38.60	1.74	27.2	25.3	6.9	20.4	Intermediate
Baseball, national from Puerto Rico	[24]	27	A [63]	24.10	1.76	24.3	15.4	3.7	20.5	Intermediate
Boxing, national from Puerto Rico	[24]	26	A [63]	18.00	1.7	22.0	11.6	2.6	19.5	Lean intermediate
Water polo, national from Puerto Rico	[24]	37	A [63]	18.00	1.75	23.9	14.3	3.4	20.5	Intermediate solid
Weightlifting, national from Puerto Rico	[24]	3	A [63]	23.70	1.66	29.9	16.3	4.9	25.1	Intermediate solid
Handball, national from Puerto Rico	[24]	14	A [63]	24.30	1.82	24.8	12.0	3.0	21.8	Lean solid
High jump, national from Puerto Rico	[24]	2	A [63]	18.00	1.85	20.3	9.0	1.8	18.5	Lean intermediate
Rowing, national from Brazil	[64]	11	A [63]	18.70	1.826	24.0	6.7	1.6	22.4	Lean solid
Basketball, European Championship 2000 Position: point guard	[65]	53	A [66]	17.80	1.88	23.0	12.2	2.8	20.2	Lean intermediate
Basketball, European Championship 2000 Position: wing	[65]	54	A [66]	17.78	1.98	23.1	13.4	3.1	20.0	Lean intermediate
Basketball, European Championship 2000 Position: pivot	[65]	25	A [66]	17.80	2.053	24.0	14.7	3.5	20.5	Intermediate
Triathlon, national from New Zealand	[67]	10	A [66]	36.20	1.777	23.6	15.1	3.6	20.0	Intermediate
Gymnastics, national from Germany	[68]	12	BIA	12.40	1.52	18.1	10.4	1.9	16.2	Lean slender
Powerlifting, national from United States Category: light weight	[69]	7	U [70]	No	1.59	25.3	13.7	3.5	21.8	Intermediate solid
Powerlifting, national from United States Category: medium weight	[69]	6	U [70]	No	1.66	28.5	14.4	4.1	24.4	Intermediate solid
Powerlifting, national from United States Category: heavy weight	[69]	7	[70]	No	1.81	41.2	26.7	11.0	30.2	Adipose solid
Bodybuilding, World Championship 2000	[71]	23	A [72]	33.42	1.66	27.2	9.7	2.6	24.6	Lean solid
Open water swimming, national from United States	[73]	4	A [74]	18.60	1.77	22.7	9.8	2.2	20.5	Lean intermediate
Artistic gymnastics, European Artistic Gymnastics Championship 2002	[75]	68	BIA	17.00	1.67	21.2	10.33	2.2	19.0	Lean intermediate
Rowing, Australian Championships 2003 Position: light weight	[76]	35	A	23.00	1.816	21.4	7.6	1.6	19.8	Lean intermediate
Rowing, Australian Championships 2003 Position: light weight	[76]	27	A	OPEN	1.8	22.0	7.3	1.6	20.4	Lean intermediate
Volleyball, national from England Position: setter	[77]	-	A [66]	17.50	1.91	19.5	12.9	2.5	17.0	Lean intermediate
Volleyball, national from England Position: opposite	[77]	-	A [66]	17.50	1.9	19.8	11.8	2.3	17.4	Lean intermediate
Volleyball, national from England Position: central	[77]	-	A [66]	17.50	1.87	22.2	11.5	2.6	19.6	Lean intermediate
Volleyball, national from England Position: attacker	[77]	-	A [66]	17.50	1.93	20.9	12.5	2.6	18.3	Lean intermediate
Rugby, national from Australia Position: defender	[78]	31	A (LifeSize computer software v1)	25.00	1.779	27.0	11.1	3.0	24.0	Lean solid
Rugby, national from Australia Position: forward	[78]	45	A (LifeSize computer software v1)	25.40	1.827	29.5	13.5	4.0	25.5	Intermediate solid
Tennis, world level	[79]	12	A [66]	16.40	1.769	22.5	15.2	3.4	19.1	Intermediate
Powerlifting, national from New Zealand Category: light weight	[80]	9	A [10] (Withers et al. unpublished)	35.40	1.63	25.9	12.1	3.1	22.8	Lean solid
Powerlifting, national from New Zealand Category: medium weight	[80]	30	A [10] (Withers et al. unpublished)	37.90	1.747	28.7	12.8	3.7	25.1	Intermediate solid
Weightlifting, national from New Zealand Category: heavy weight	[80]	15	A [10] (Withers et al. unpublished)	33.40	1.747	39.9	26.4	10.5	29.4	Adipose solid
Judo, national from Brazil	[81]	22	A [63]	25.60	1.762	29.2	11.4	3.3	25.9	Intermediate solid
Handball, 12th Asian Games, England’s team	[82]	8	A [66]	20.00	1.74	25.6	13.4	3.4	22.2	Intermediate solid
Handball, 12th Asian Games, China’s team	[82]	10	A [66]	25.00	1.9	23.7	9.6	2.3	21.4	Lean intermediate
Handball, 12th Asian Games, Japan’s team	[82]	16	A [66]	26.00	1.85	23.6	9.2	2.2	21.4	Lean intermediate
Handball, 12th Asian Games, Korea’s team	[82]	7	A [66]	25.00	1.84	25.2	11.2	2.8	22.4	Lean solid
Handball, 12th Asian Games, Kuwait’s team	[82]	17	A [66]	26.00	1.81	26.7	12.9	3.4	23.3	Intermediate solid
Handball, 12th Asian Games, Saudi Arabia’s team	[82]	13	A [66]	25.00	1.82	22.9	10.3	2.4	20.5	Lean intermediate
Ultratriathlon, World Championship	[83]	1	BIA	43.00	1.78	25.1	13.40	3.4	21.7	Intermediate solid
Bouldering, world class	[84]	18	A [63]	25.80	1.74	22.2	5.8	1.3	20.9	Lean intermediate
Windsurfing, World Championship	[85]	15	A [10] (Withers et al. unpublished)	25.40	1.846	24.4	10.7	2.6	21.8	Lean solid
Judo, national from Spain	[86]	8	A [87]	22.10	1.798	25.5	8.0	2.0	23.5	Lean solid
Flatwater kayak, national	[88]	28	A [89]	16.30	1.756	23.7	22.7	5.4	18.3	Intermediate
Flatwater kayak, national	[88]	27	A [89]	23.70	1.769	25.2	23.7	6.0	19.2	Intermediate
Greco-Roman wrestling, medalists of international tournaments	[90]	12	A [63]	22.10	1.74	23.8	7.6	1.8	22.0	Lean solid
Marathon, national from Switzerland	[91]	81	A [92]	43.30	1.8	22.6	16.2	3.7	18.9	Intermediate
Mountaineering, 21 years of experience	[93]	10	A [66]	41.40	1.7612	22.7	11.8	2.7	20.1	Lean intermediate
Marathon, high level, Kenya	[94]	14	A [63]	27.71	1.7121	19.7	8.9	1.7	17.9	Lean intermediate
Pentathlon, World Championship	[95]	7	BIA	19.60	1.847	21.4	8.8	1.9	19.5	Lean intermediate
Greco-Roman wrestling, Olympic level	[96]	21	A [63]	27.90	1.656	24.2	13.6	3.3	20.9	Intermediate
Professional surfing, International World Qualifying Series 5-Star event 2010	[97]	17	A [87]	34.12	1.77	25.1	11.3	2.8	22.3	Lean solid
Triathlon, Spanish university championship	[98]	39	A [10] (Withers et al. unpublished)	24.00	1.77	22.6	10.2	2.3	20.2	Lean intermediate
Tennis, national university championship	[99]	26	A [10] (Withers et al. unpublished)	23.00	1.8	23.1	16.2	3.7	19.3	Intermediate
Paddle, national university championship	[99]	21	A [10] (Withers et al. unpublished)	23.10	1.8	22.9	18.3	4.2	18.7	Intermediate
Men’s Physique, national competition from England	[100]	1	A [66]	21.00	1.785	27.0	14.0	3.8	23.2	Intermediate solid
Basque pelota, Spain national	[101]	8	A [87]	25.30	1.83	25.7	8.9	2.3	23.4	Lean solid
Basque pelota, international championship	[101]	10	A [87]	22.80	1.775	25.5	11.6	3.0	22.5	Lean solid
Baseball, honor division Position: pitcher	[102]	-	A [103]	23.87	1.79	26.1	23.88	6.2	19.9	Intermediate
Baseball, honor division Position: catcher	[102]	-	A [103]	23.87	1.82	25.2	23.85	6.0	19.2	Intermediate
Baseball, honor division Position: first base	[102]	-	A [103]	23.87	1.82	25.6	22.47	5.8	19.9	Intermediate
Baseball, honor division Position: second base	[102]	-	A [103]	23.87	1.81	26.0	23.21	6.0	20.0	Intermediate
Baseball, honor division Position: Third base	[102]	-	A [103]	23.87	1.82	25.9	22.67	5.9	20.1	Intermediate
Baseball, honor division Position: shortstop	[102]	-	A [103]	23.87	1.82	25.5	22.95	5.8	19.6	Intermediate
Baseball, honor division Position: left fielder	[102]	-	A [103]	23.87	1.81	26.1	22.89	6.0	20.1	Intermediate
Baseball, honor division Position: central fielder	[102]	-	A [103]	23.87	1.81	25.6	23.86	6.1	19.5	Intermediate
Baseball, honor division Position: right fielder	[102]	-	A [103]	23.87	1.82	25.7	23.59	6.1	19.7	Intermediate
Brazilian Jiu-Jitsu, Brazil national and international medalists	[104]	8	A [105]	25.00	1.72	25.7	11.2	2.9	22.8	Lean solid
Judo, national from Spain	[106]	9	A [87]	20.00	1.8	26.5	7.8	2.1	24.4	Lean solid
Sprinters 100 m from Italy and Croatia	[107]	98	A [63]	23.10	1.77	23.3	7.7	1.8	21.5	Lean intermediate
Open water swimming, 2015 World Championships	[108]	4	A [10] (Withers et al. unpublished)	24.00	1.83	24.8	11.5	2.8	21.9	Lean solid
Open water swimming, 2015 World Championships	[108]	4	A [10] (Withers et al. unpublished)	21.70	1.85	21.1	9.4	2.0	19.1	Lean intermediate
Basketball, national from Poland	[109]	35	BIA	24.45	1.9344	24.1	14.0	3.4	20.7	Intermediate
Mountain biking, Olympic	[110]	5	A [66]	30.30	1.78	22.2	9.9	2.2	20.0	Lean intermediate
Mountain biking, world champion	[110]	22	A [66]	28.00	1.75	21.9	9.5	2.1	19.8	Lean intermediate
Racewalking, Pan American Games	[111]	10	A [87]	17.20	1.71	20.6	7.64	1.6	19.1	Lean intermediate
Powerlifting, national from Chile Category: light weight	[112]	18	A	27.20	1.68	25.0	16.2	4.1	21.0	Intermediate
Powerlifting, national from Chile Category: medium weight	[112]	12	A	35.00	1.747	28.5	18.2	5.1	23.3	Intermediate solid
Powerlifting, national from Chile Category: heavy weight	[112]	10	A	31.00	1.726	36.2	31.0	11.2	24.9	Intermediate solid
Soccer, national from Bosnia and Herzegovina	[113]	28	BIA	24.36	1.82	23.8	8.79	2.1	21.7	Lean solid
Soccer, national from Montenegro	[113]	30	BIA	22.73	1.81	23.8	9.98	2.4	21.4	Lean intermediate
Long-distance runners, national from Brazil (population: military)	[114]	17	A [63]	23.70	1.75	21.3	4.7	1.0	20.3	Lean intermediate
Bouldering, international Level: 8b (Fontainebleau Scale)	[115]	11	BIA	26.30	1.74	21.4	7.4	1.6	19.8	Lean intermediate
Lead climbing, international Level: 8c (Fontainebleau Scale)	[115]	8	BIA	22.60	1.74	20.5	8.0	1.6	18.9	Lean intermediate
Speed climbing international Level: 15 m high with a fall angle of 5° in 6.28 s	[115]	5	BIA	24.20	1.77	22.0	9.4	2.1	19.9	Lean intermediate
Soccer, first national division from Spain Position: lateral	[116]	10	A [87]	21.30	1.76	22.3	7.0	1.6	20.7	Lean intermediate
Soccer, first national division of Spain Position: lateral defender	[116]	12	A [87]	21.30	1.75	23.1	6.8	1.6	21.6	Lean solid
Soccer, first national division of Spain Position: central defender	[116]	7	A [87]	21.30	1.85	23.0	6.8	1.6	21.4	Lean intermediate
Soccer, first national division of Spain Position: defender	[116]	19	A [87]	21.30	1.78	23.3	6.8	1.6	21.7	Lean intermediate
Soccer, first national division of Spain Position: midfielder	[116]	23	A [87]	21.30	1.77	22.5	7.0	1.6	20.9	Lean intermediate
Soccer, first national division of Spain Position: central midfielder	[116]	13	A [87]	21.30	1.77	22.9	7.1	1.6	21.3	Lean intermediate
Soccer, first national division of Spain Position: Forward	[116]	7	A [87]	21.30	1.84	22.8	7.34	1.7	21.1	Lean intermediate
Rowing, national from Spain Position: light weight	[117]	5	BIA	23.00	1.79	23.5	12.3	2.9	20.6	Lean intermediate
Rowing, national from Spain Position: heavy weight	[117]	11	BIA	23.00	1.9	24.6	15.1	3.7	20.9	Intermediate
Sprint swimming, international	[118]	46	BIA	22.90	1.86	23.8	9.82	2.3	21.5	Lean intermediate
Cycling, national from Italy	[119]	8	BIA	19.38	1.75	21.9	16.07	3.5	18.4	Intermediate
Cycling, national from Italy	[119]	8	BIA	19.38	1.79	22.6	15.95	3.6	19.0	Intermediate
Soccer, first national division of Colombia Position: goalkeeper	[120]	2	BIA	21.00	1.85	23.6	12.6	3.0	20.7	Lean intermediate
Soccer, first national division of Colombia Position: defender	[120]	5	BIA	21.00	1.81	23.0	13	3.0	20.0	Lean intermediate
Soccer, first national division of Colombia Position: midfielder	[120]	13	BIA	21.00	1.75	22.8	14.4	3.3	19.5	Intermediate
Soccer, first national division of Colombia Position: forward	[120]	4	BIA	21.00	1.73	25.5	15.2	3.9	21.6	Intermediate solid
Rowing, national from Spain	[121]	13	A [10] (Withers et al. unpublished)	27.30	1.82	22.7	10.3	2.3	20.4	Lean intermediate
Taekwondo, at the national and international level Category: <54 kg	[122]	8	A [10] (Withers et al. unpublished)	20.20	1.74	18.6	10.4	1.9	16.7	Lean intermediate
Taekwondo, at the national and international level Category: <58 kg	[122]	8	A [10] (Withers et al. unpublished)	19.14	1.78	19.0	10.6	2.0	17.0	Lean intermediate
Taekwondo, at the national and international level Category: <63 kg	[122]	8	A [10] (Withers et al. unpublished)	21.19	1.78	20.5	10.9	2.2	18.3	Lean intermediate
Taekwondo, at the national and international level Category: <68 kg	[122]	14	A [10] (Withers et al. unpublished)	22.42	1.81	21.7	11.2	2.4	19.2	Lean intermediate
Taekwondo, at the national and international level Category: <74 kg	[122]	9	A [10] (Withers et al. unpublished)	22.83	1.83	23.0	12.1	2.8	20.2	Lean intermediate
Taekwondo, at the national and international level Category: <80 kg	[122]	7	A [10] (Withers et al. unpublished)	21.24	1.87	23.6	12.9	3.0	20.5	Lean intermediate
Taekwondo, at the national and international level Category: >87 kg	[122]	5	A [10] (Withers et al. unpublished)	27.18	1.87	26.1	13.2	3.4	22.7	Lean intermediate
Sprinters, at the national university level from Poland	[123]	26	BIA	20.37	1.8	22.9	17.06	3.9	19.0	Intermediate
Middle distance runners, at the national university level from Poland	[123]	22	BIA	20.31	1.81	21.1	18.71	4.0	17.2	Intermediate
Long distance runners, at the national university level from Poland	[123]	20	BIA	21.39	1.77	21.6	18.91	4.1	17.5	Intermediate

Abbreviations: A: anthropometry; BF%: body fat percentage; BIA: bioelectrical impedance; FFMI: fat-free mass index; FMI: fat mass index; BMI: body mass index; and U: ultrasound. FMI and FFMI are derived from height-adjusted values. BF% values were extracted from original studies when available. When BF% was not reported, values were estimated from the fat mass or lean mass provided in the article using proportional calculations based on the reported components. A study did not report sample size by position [102].

**Table 6:** Body composition in elite female athletes with doubly indirect methods.

Sport	Reference	*n*	Method	Age, Years	Height, m	BMI, kg/m^2^	BF%	FMI, kg/m^2^	FFMI, kg/m^2^	Body Physique
Archery, national from Puerto Rico	[24]	4	A [124]	24.00	1.56	22.9	26.7	6.1	16.8	Intermediate
Bowling, national from Puerto Rico	[24]	19	A [124]	27.20	1.59	25.9	32.1	8.3	17.6	Intermediate
Road cycling, national from Puerto Rico	[24]	8	A [124]	23.70	1.62	21.1	19.4	4.1	17.0	Lean intermediate
Fencing, national from Puerto Rico	[24]	11	A [124]	17.40	1.59	23.0	22.1	5.1	17.9	Intermediate
Sport shooting, national from Puerto Rico	[24]	4	A [124]	44.70	1.6	26.2	33.5	8.8	17.4	Intermediate
Softball, national from Puerto Rico	[24]	12	A [124]	24.90	1.62	23.7	33.9	8.0	15.7	Intermediate
Bodybuilding, national from Brazil	[125]	11	A [124]	28.70	1.619	21.3	8.1	1.7	19.6	Lean solid
Rowing, Brazil national team	[64]	4	A [124]	18.70	1.722	21.8	19.9	4.3	17.4	Lean intermediate
Triathlon, national from New Zealand	[67]	8	A [124]	34.30	1.664	22.5	22.2	5.0	17.5	Intermediate
Gymnastics, national from Germany	[126]	15	BIA	13.60	1.5	18.1	14.4	2.6	15.5	Lean intermediate
Soccer, high level from Turkey	[127]	17	A [124]	20.73	1.624	21.5	19.8	4.2	17.2	Lean intermediate
Ballet, 15 years of experience	[128]	10	BIA	19.20	1.647	19.6	19.9	3.9	15.7	Lean intermediate
Modern ballet, 13 years of experience	[128]	7	BIA	22.00	1.633	20.5	19.3	4.0	16.6	Lean intermediate
Open water swimming, national from United States	[73]	4	A [124]	17.80	1.68	22.5	22.8	5.1	17.4	Intermediate
Artistic gymnastics, European Artistic Gymnastics Championship 2002	[75]	120	BIA	15.00	1.51	18.9	18.37	3.5	15.4	Lean intermediate
Rowing, Australian Championships 2003 Position: light weight	[76]	28	A	23.00	1.7	19.9	12.4	2.5	17.4	Lean intermediate
Rowing, Australian Championships 2003 Position: light weight	[76]	17	A	OPEN	1.7	20.0	11.74	2.4	17.7	Lean intermediate
Volleyball, national from Greece	[129]	163	A [89]	23.80	1.771	22.2	23.4	5.2	17.0	Intermediate
Basketball, national from Greece	[129]	133	A [89]	22.10	1.747	23.4	24.3	5.7	17.7	Intermediate
Handball, national from Greece	[129]	222	A [89]	21.50	1.659	23.7	25.9	6.1	17.5	Intermediate
Tennis, world class	[79]	45	A [89]	16.10	1.7	21.5	28.6	6.1	15.3	Intermediate
Water polo, national from Scotland	[130]	14	A [89]	22.00	1.71	22.7	23.1	5.2	17.4	Intermediate
Soccer, first national division from Spain	[131]	100	A [72]	22.10	1.613	22.2	20.1	4.5	17.7	Lean intermediate
Bouldering, world class	[84]	7	A [124]	25.10	1.62	20.6	16.6	3.4	17.2	Lean intermediate
Judo, Spain, national level	[86]	18	A [87]	24.10	1.633	24.3	19.9	4.8	19.4	Intermediate solid
Flatwater kayak, national from Greece, junior	[88]	15	A [89]	16.20	1.671	21.1	25.9	5.5	15.7	Intermediate
Flatwater kayak, national from Greece	[88]	13	A [89]	22.70	1.658	24.0	26.4	6.3	17.6	Intermediate
Pentathlon, World Championship	[95]	5	BIA	17.40	1.707	21.5	15.8	3.4	18.1	Intermediate solid
Volleyball, national from Spain Position: setter	[132]	29	A [66]	24.80	1.76	22.0	21.8	4.8	17.2	Intermediate
Volleyball, national from Spain Position: opposite	[132]	18	A [66]	24.80	1.83	24.4	25.2	6.2	18.3	Intermediate solid
Volleyball, national from Spain Position: central	[132]	47	A [66]	24.80	1.84	22.4	23	5.2	17.3	Intermediate
Volleyball, national from Spain Position: attacker	[132]	41	A [66]	24.80	1.83	22.9	23.3	5.3	17.6	Intermediate
Volleyball, national from Spain Position: libero	[132]	13	A [66]	24.80	1.71	23.6	24.8	5.8	17.7	Intermediate
Volleyball, CEV European Championship Cup champion, MEVZA League champion	[133]	18	BIA	27.40	1.843	21.2	19.8	4.2	17.0	Lean intermediate
Soccer, Czech Republic team participation in the European Championship	[133]	18	BIA	23.20	1.673	21.9	19.5	4.3	17.6	Lean intermediate
Basketball, Czech Republic team silver medal at the World Championship	[133]	14	BIA	25.90	1.858	22.2	21.2	4.7	17.5	Intermediate
Handball, Czech Republic team	[133]	16	BIA	24.00	1.76	23.4	21.4	5.0	18.4	Intermediate solid
Softball, participation in the World Championship	[133]	14	BIA	23.60	1.699	23.5	21.4	5.0	18.5	Intermediate solid
Cycling, world class	[134]	32	A [135]	27.00	1.7	20.6	14.5	3.0	17.6	Lean intermediate
Cycling, national from Australia	[134]	60	A [135]	25.60	1.693	21.0	15.5	3.3	17.7	Lean intermediate
Judo, national from Spain	[106]	17	A [87]	20.00	1.64	23.2	17.0	3.9	19.3	Lean solid
Open water swimming, World Championships 2015	[108]	4	A [135]	23.40	1.71	22.7	20.5	4.6	18.0	Intermediate
Open water swimming, World Championships 2015	[108]	3	A [135]	23.00	1.67	20.5	13.5	2.8	17.7	Lean solid
Racewalking, Pan American Games	[111]	20	A [87]	19.45	1.63	20.8	13.94	2.9	17.9	Lean intermediate
Canoe polo, national from Italy	[136]	21	BIA	26.80	1.66	22.3	21	4.7	17.6	Intermediate
Powerlifting, national from Chile Category: light weight	[112]	8	A	28.30	1.543	20.7	21.3	4.4	16.3	Lean intermediate
Powerlifting, national from Chile Category: medium and heavy weight	[112]	8	A	26.00	1.643	23.6	26.3	6.2	17.4	Intermediate
Sprinters 100 m, Olympic	[137]	5	U [70]	21.20	1.6	19.9	14.93	3.0	16.9	Lean intermediate
Sprinters 100 m hurdles, Olympic	[137]	2	U [70]	21.20	1.6	20.1	16.44	3.3	16.8	Lean intermediate
Sprinters 400 m, Olympic	[137]	5	U [70]	21.20	1.6	20.7	15.92	3.3	17.4	Lean intermediate
Professional cheerleaders	[138]	19	BIA	25.40	1.64	21.4	22.2	4.7	16.6	Intermediate
Football, national from Brazil	[139]	115	A [124]	22.00	1.61	22.6	22.1	5.0	17.6	Intermediate
Rowing, national level from Spain Category: light weight	[117]	2	BIA	23.00	1.69	22.0	20	4.4	17.6	Lean intermediate
Rowing, national level from Spain Category: heavy weight	[117]	2	BIA	23.00	1.78	23.5	25.7	6.0	17.5	Intermediate
Sprint swimming, international	[118]	36	BIA	21.00	1.73	21.0	15.79	3.3	17.7	Lean intermediate
Rowers, national from Spain	[121]	11	A [135]	27.70	1.69	21.7	15.4	3.3	18.3	Lean solid

Abbreviations: A: anthropometry; BF%: body fat percentage; BIA: bioelectrical impedance; BMI: body mass index; CEV: Confédération Européenne de Volleyball; FMI: fat mass index; FFMI: fat-free mass index; MEVZA: Middle European Volleyball Zonal Association; and U: ultrasound. FMI and FFMI are derived from height-adjusted values. BF% values were extracted from original studies when available. When BF% was not reported, values were estimated from the fat mass or lean mass provided in the article using proportional calculations based on the reported components.

**Table 7:** Description of body physique categories measured with indirect methods in elite athletes.

Males	F	BMI		BF%		FMI		FFMI		Athletes
		Mean ± SD	Interval	Mean ± SD	Interval	Mean ± SD	Interval	Mean ± SD	Interval	
Adipose solid	4	37.1 ± 0.7	36.5–37.9	26.8 ± 1.6	25.1–28.8	9.9 ± 0.7	9.2–10.9	27.1 ± 0.3	26.9–28.2	LS, LO, LD
Intermediate	2	23.3 ± 1.0	22.3–24.3	16.2 ± 1.2	14.9–17.4	3.7 ± 0.4	3.3–4.2	19.5 ± 0.5	19.0–20.1	TD, LRC
Intermediate solid	18	30.1 + 3.4	26.1–43.7	15.9 + 2.6	12.1–19.6	4.8 ± 1.2	3.3–8.2	25.2 ± 2.5	22.3–35.5	MC, AC, A, LD, W, JR, P, R, JC, A, PA, J, AF
Lean intermediate	10	20.1 ± 1.2	20.1–24.3	11.8 ± 1.3	8.8–13.9	2.6 ± 0.3	1.8–3.2	19.7 ± 1.0	17.5–21.1	J, E, JP, JS, BS, B, LUO, TA, FU
Lean solid	9	25.1 ± 1.4	22.9–29.8	9.1 ± 2.3	3.4–12.2	2.3 ± 0.6	0.8–3.0	22.8 ± 1.4	21.1–27.6	FU, C, R, DT, PA, BL, FA, JR
**Females**	**F**	**BMI**		**BF%**		**FMI**		**FFMI**		**Athletes**
		Mean ± SD	Interval	Mean ± SD	Interval	Mean ± SD	Interval	Mean ± SD	Interval	
Intermediate	6	22.6 ± 0.5	21.2–23.2	23.5 ± 0.9	22.0–25.1	5.3 ± 0.2	4.9–5.8	17.2 ± 0.4	16.3–17.6	B, TA, JL, N, BA
Intermediate solid	4	25.0 ± 0.8	23.9–26.2	25.2 ± 3.2	21.6–29.7	6.3 ± 1.0	5.3–7.8	18.7 ± 0.4	18.4–19.4	LUO, BA, J
Intermediate solid	7	20.2 ± 0.9	17.5–21.7	20.9 ± 1.7	15.9–22.6	4.1 ± 0.6	2.3–4.5	16.0 ± 0.7	14.7–17.3	BS, TD, BL, BAL, G, CC, FU
Lean solid	1	21.6 ± 0	21.6	9.7 ± 0	9.7	2.1 ± 0	2.1	19.5 ± 0	19.5	FI

Abbreviations: LS: sumo fighter; LO: lineman offensive; LD: lineman defensive; TD: ten dance; LRC: cricket fast bowler; MC: field marshal; AC: tight end; A: supporter; W: water polo player; JR: rugby player; P: deeper; R: receptor; JC: runner; PA: kicker; J: judo player; AF: strength athlete; E: fencer; JP: track rider; JS: jump rider; BS: standard dancer; B: boxer; LUO: Olympic fighter; TA: taekwondo athlete; FU: football player; BL: Latin dancer; FA: Australian football player; JL: lacrosse player; N: swimmer; BA: hand ball player; BAL: ballet; FI: bodybuilder; G: gymnastics; CC: field runner; FU: football player; F: frequency; BMI: body mass index; BF%: body fat; FMI: fat mass index; FFMI: fat-free mass index; and SD: standard deviation.

**Table 8:** Description of body physique categories measured with doubly indirect methods in elite athletes.

Males	F	BMI		BF%		FMI		FFMI		Athletes
		Mean ± SD	Interval	Mean ± SD	Interval	Mean ± SD	Interval	Mean ± SD	Interval	
Adipose solid	3	39.0 ± 2.0	36.2–41.2	27.9 ± 2.1	26.4–31.0	10.8 ± 0.3	10.5–11.2	28.1 ± 2.2	24.9–30.2	LPP
Intermediate	31	24.1 ± 1.5	21.1–27.2	19.2 ± 3.8	13.6–25.3	4.7 ± 1.1	3.3–6.9	19.5 ± 0.9	17.2–21.0	LUO, BAS, TE, PAD, TR, LPL, K, BEI, FU, V, C, R, CY, A, BO, TD
Intermediate solid	14	27.4 ± 2.1	23.9–29.9	13.6 ± 1.5	11.4–18.0	3.7 ± 0.4	3.3–5.2	23.7 ± 1.9	20.5–25.9	LPM, JR, MP, UL, BA, FU, LPL, TA, W, LP, J
Lean slender	1	18.1 ± 0	18.1	10.4 ± 0	10.4	1.9 ± 0	1.9	16.2 ± 0	16.2	G
Lean intermediate	49	22.2 ± 1.1	18.6–23.8	9.8 ± 2.1	4.7–14.1	2.1 ± 0.5	1.0–3.2	20.0 ± 1.0	16.7–21.7	RE, MA, TR, VO, BAS, FU, PEN, AL, BA, V, C, CM, AM, EB, NAA, G, NV, TA, CR, E, B, SA, EP, EV
Lean solid	17	25.2 ± 1.2	23.1–27.2	9.8 ± 1.7	6.7–12.1	2.5 ± 0.4	1.6–3.1	22.7 ± 1.0	21.6–24.6	CA, J, FI, JR, LPL, FU, WIN, PE, BA, LUG, LUJ, NAA, S
**Females**	**F**	**BMI**		**BF%**		**FMI**		**FFMI**		**Athletes**
		Mean ± SD	Interval	Mean ± SD	Interval	Mean ± SD	Interval	Mean ± SD	Interval	
Intermediate	25	22.9 ± 0.8	21.1–26.2	24.4 ± 2.4	20.5–33.9	5.5 ± 0.7	4.6–8.8	17.2 ± 0.5	15.3–18.0	VO, BAS, TE, TR, W, K, LP, FUT, NAA, JPC, PO, AR, BO, E, TD, SO
Lean solid	4	23.9 ± 0.4	23.4–24.4	22.0 ± 2.0	19.9–25.2	5.2 ± 0.5	4.8–6.2	18.6 ± 0.4	18.3–19.4	J, BA, SO, VO
Lean intermediate	23	20.5 ± 1.2	18.1–22.2	17.3 ± 2.6	11.7–21.3	3.5 ± 0.6	2.4–4.5	16.9 ± 0.9	15.4–17.9	R, FU, CA, CY, BAL, VO, LP, G, AM, EB, NAA, NV, CR
Lean solid	4	22.1 ± 0.8	21.3–23.2	14.2 ± 3.6	8.1–17.0	3.1 ± 0.8	1.7–3.9	18.9 ± 0.5	18.1–19.6	FI, J, PEN, R

Abbreviations: LPP: heavy-class powerlifter; LUO: Olympics fighter; BAS: basketball player; TE: tennis player; PAD: padel player; TR: triathlete; LPL: light-class powerlifter; K: kayak; BEI: baseball player; FU: football player; V: sprinter; C: runner; R: rower; CY: cycling; A: archery; BO: bowl player; TD: shooting; LPM: medium-class powerlifter; JR: rugby player; MP: male body physique; UL: ultra triathlete; BA: handball player; TA: taekwondo player; W: water polo player; LP: powerlifter; G: gymnastics; M: marathon runner; VO: volleyball player; PEN: pentathlete; AL: mountaineer; CM: mountaineer cyclist; AM: walking athlete; EB: bouldering climber; NAA: open water swimmer; NV: speed swimmer; CR: road cyclist; E: fencer; B: boxer; SA: high jump athlete; EP: top climber; EV: speed climber; CA: canoe; J: judo player; WIN: windsurf player; PE: pelota player; LUG: Greco-Roman fighter; LUJ: jiu-jitsu fighter; S: surf; FUT: futsal player; JPC: canoe polo player; PO: cheerleader; ESO: softball; FI: body builder; BAL: ballet; F: frequency; BMI: body mass index; BF%: body fat; FMI: fat mass index; FFMI: fat-free mass index; and SD: standard deviation.

## Data Availability

The original contributions presented in this study are included in the article. Further inquiries can be directed to the corresponding author(s).

## Supplementary Materials

The following supporting information can be downloaded at: https://www.mdpi.com/article/10.3390/jfmk11010013/s1, Figure S1: Body composition Fat-free mass index vs. fat mass index) in elite male athletes measured with indirect methods. Figure S2. Body composition Fat-free mass index vs. fat mass index) in elite female athletes measured with indirect methods. Figure S3. Body composition Fat-free mass index vs. fat mass index) in elite male athletes measured with doubly indirect methods. Figure S4. Body composition Fat-free mass index vs. fat mass index) in elite female athletes measured with doubly indirect methods.
