# Supplementary figures and images for: Defining Elite Zones: A Scoping Review of Body Physique and Body Fat in Elite Athletes
Source: J Funct Morphol Kinesiol. 2025 Dec 29;11(1):13. doi: 10.3390/jfmk11010013 (PMC12821704; doi:10.3390/jfmk11010013)

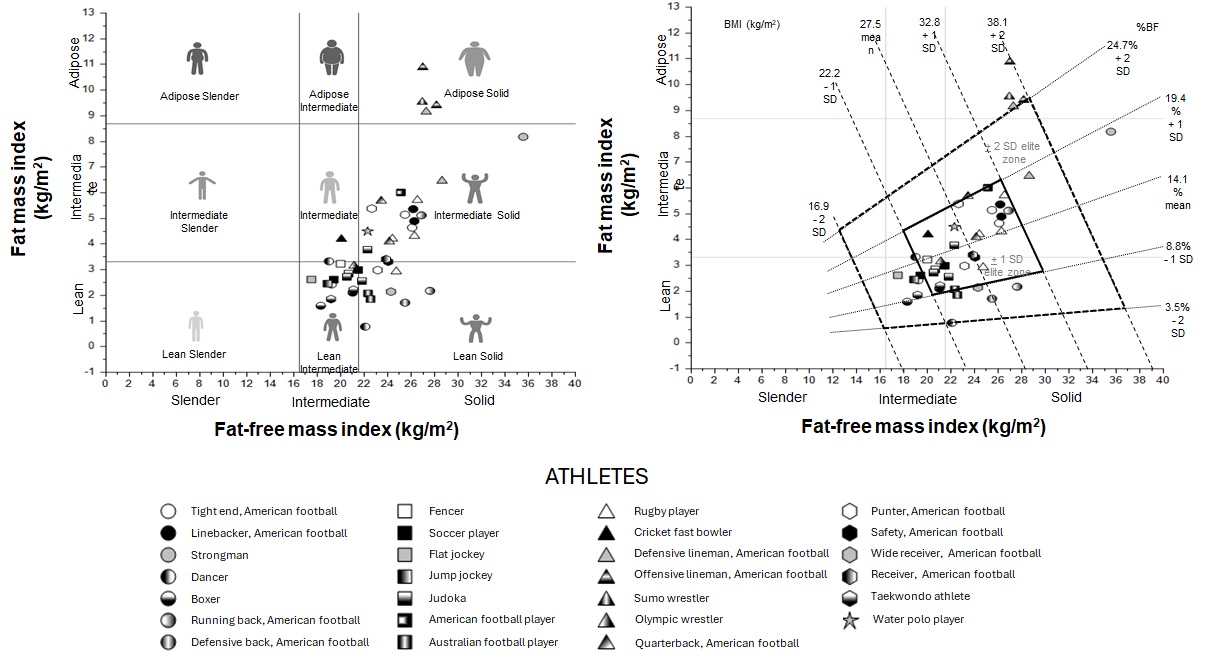

Supplement: Supplementary file 1 [file jfmk-11-00013-s001.zip › Figure S1.jpg]

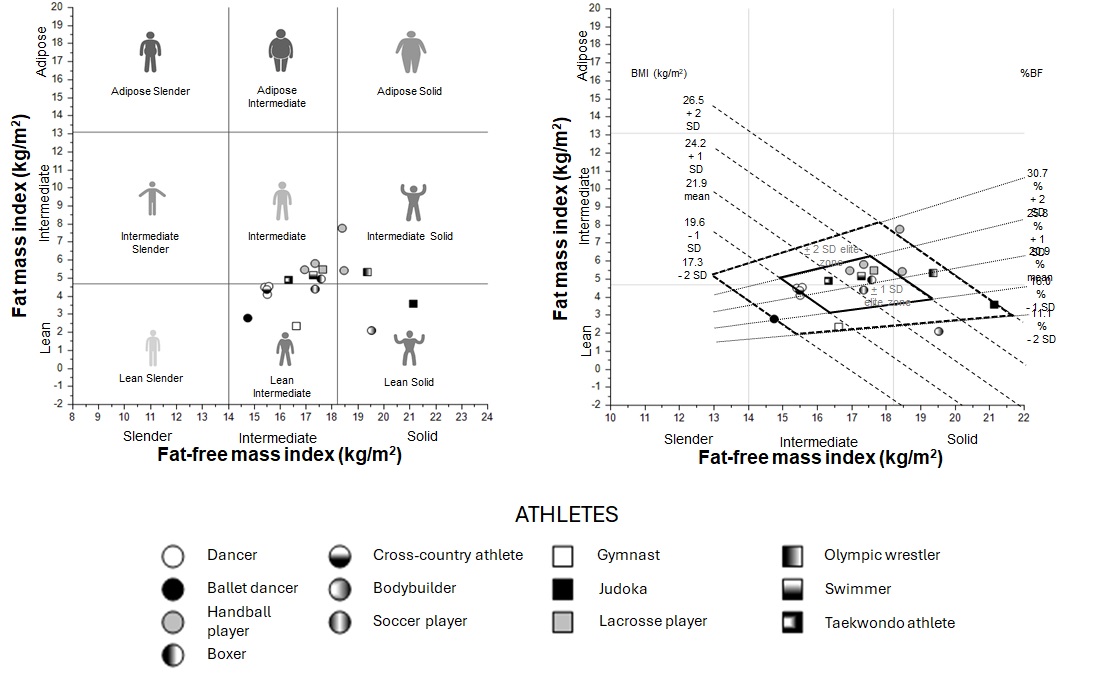

Supplement: Supplementary file 1 [file jfmk-11-00013-s001.zip › Figure S2.jpg]

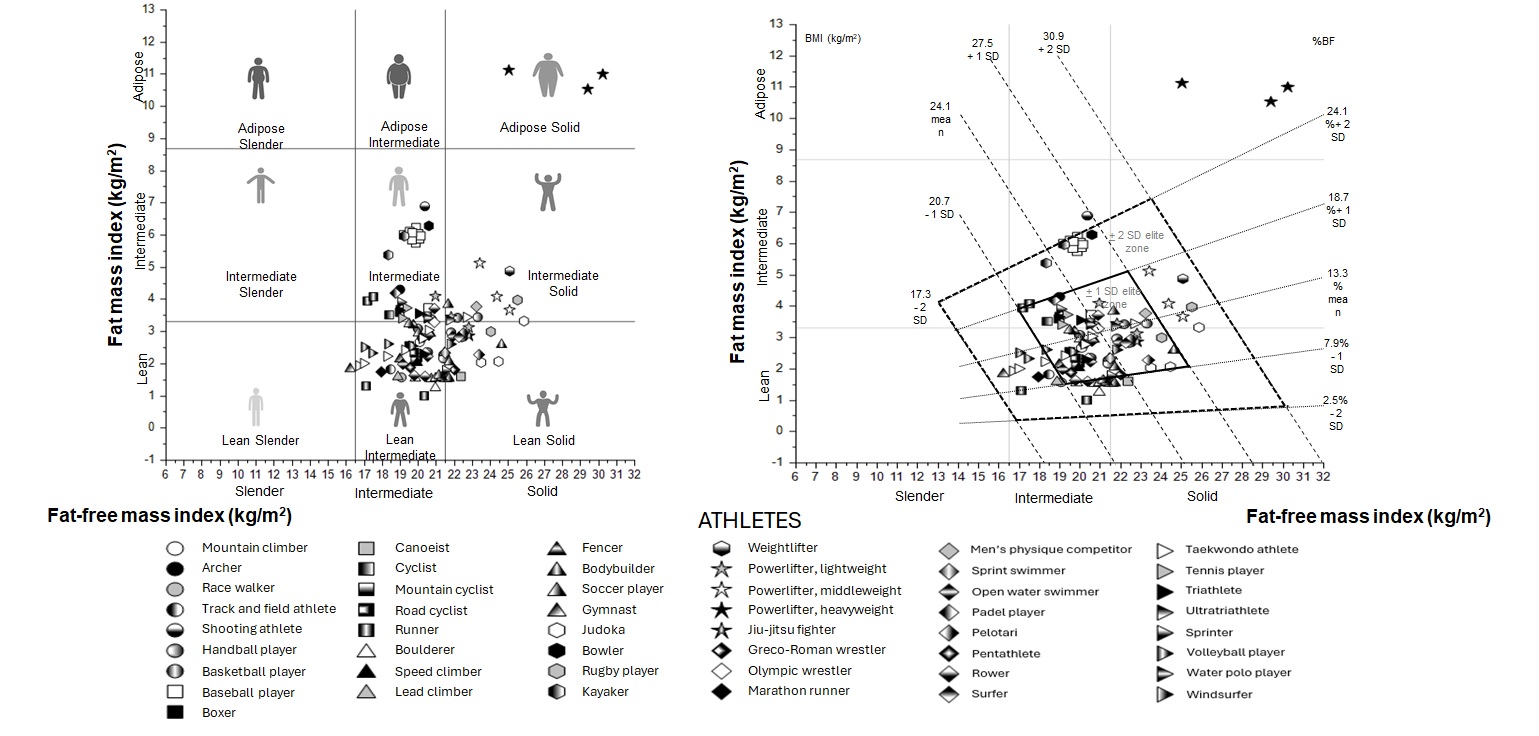

Supplement: Supplementary file 1 [file jfmk-11-00013-s001.zip › Figure S3.jpg]

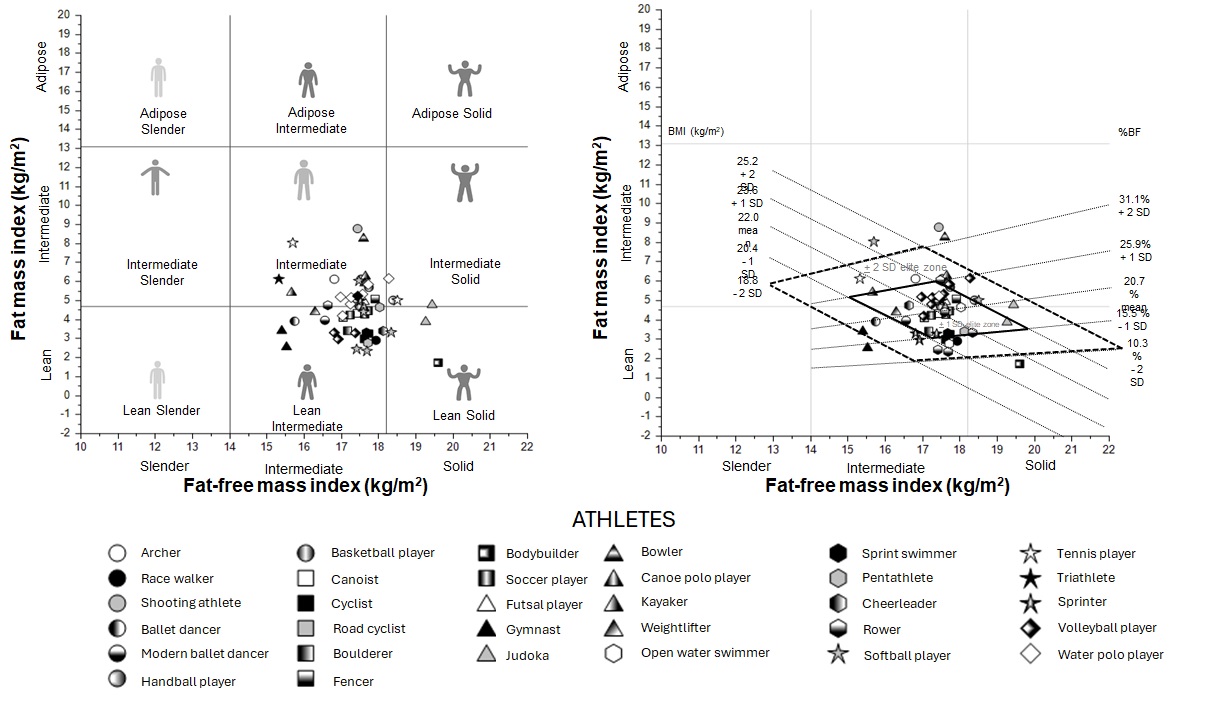

Supplement: Supplementary file 1 [file jfmk-11-00013-s001.zip › Figure S4.jpg]
